# Estradiol Facilitation of Cocaine Self-Administration in Female Rats Requires Activation of mGluR5

**DOI:** 10.1523/ENEURO.0140-16.2016

**Published:** 2016-10-25

**Authors:** Luis A. Martinez, Kellie S. Gross, Brett T. Himmler, Nicole L. Emmitt, Brittni M. Peterson, Natalie E. Zlebnik, M. Foster Olive, Marilyn E. Carroll, Robert L. Meisel, Paul G. Mermelstein

**Affiliations:** 1 Department of Neuroscience, University of Minnesota, Minneapolis, MN 55455; 2 Graduate Program in Neuroscience, University of Minnesota, Minneapolis, MN 55455; 3 College of Veterinary Medicine and Doctor of Veterinary Medicine Program, University of Minnesota, Minneapolis, MN 55455; 4 Department of Anatomy and Neurobiology, University of Maryland School of Medicine, Baltimore, Maryland, 21201; 5 Department of Psychology and Interdisciplinary Graduate Program in Neuroscience, Arizona State University, Tempe, AZ 85287; 6 Department of Psychiatry, University of Minnesota, Minneapolis, MN 55455

**Keywords:** drug addiction, estrogen, glutamate, nucleus accumbens, plasticity, psychostimulant

## Abstract

In comparison to men, women initiate drug use at earlier ages and progress from initial use to addiction more rapidly. This heightened intake and vulnerability to drugs of abuse is regulated in part by estradiol, although the signaling mechanisms by which this occurs are not well understood. Recent findings indicate that within the nucleus accumbens core, estradiol induces structural plasticity via membrane-localized estrogen receptor α, functionally coupled to metabotropic glutamate receptor subtype 5 (mGluR5). Hence, we sought to determine whether mGluR5 activation was essential for estradiol-mediated enhancement of cocaine self-administration. Ovariectomized (OVX) female rats were allowed to freely self-administer cocaine under extended access conditions (6 h/d) for 10 consecutive days. The mGluR5 antagonist 2-methyl-6-(phenylethynyl)pyridine hydrochloride (MPEP) or vehicle was administered before estradiol (or oil), on a 2 d on/2 d off schedule throughout the extended access period. MPEP treatment prevented the estradiol-dependent enhancement of cocaine self-administration in OVX females. In a separate experiment, potentiation of mGluR5 function with the positive allosteric modulator 3-cyano-*N*-(1,3-diphenyl-1H-pyrazol-5-yl)benzamide (in the absence of estradiol treatment) failed to increase cocaine self-administration. These data suggest that mGluR5 activation is necessary for estradiol-mediated enhancement of responses to cocaine, but that direct mGluR5 activation is insufficient to mimic the female response to estradiol. Building on previous studies in male animals, these findings further highlight the therapeutic potential of mGluR5 antagonism in the treatment of addiction and suggest that there may be added therapeutic benefit in females.

## Significance Statement

Gonadal steroid hormones, including estradiol, contribute to the enhanced progression of drug addiction in women. The mechanisms responsible for this effect, however, remain poorly understood. Here we show that activation of the group I metabotropic glutamate receptor subtype 5 (mGluR5) is required for the facilitative effects of estradiol on cocaine self-administration in ovariectomized female rats. Given recent work demonstrating that the estradiol-mGluR5 signaling is found only in females, the present findings suggest that pharmacological blockade of mGluR5 may have particular therapeutic potential for treating addiction in women.

## Introduction

Although drug addiction affects both sexes, addiction develops and progresses more rapidly in females compared with males. Specifically, women start using various drugs of abuse, including psychostimulants, at an earlier age than men, and as a result reach clinical stages of addiction more quickly following initial use (Quinones-Jenab and Jenab, 2012). This sex difference in the progression of addiction appears to be driven by enhanced sensitivity to drugs of abuse in women. Indeed, women report a greater subjective high in response to cocaine, even when drug levels and metabolite production are equivalent across sexes (Griffin et al., 1989; McCance-Katz et al., 2005). The subjective responses to drugs of abuse in women fluctuate across the reproductive cycle (Evans et al., 2002), suggesting that gonadal sex steroid hormones may contribute to the observed sex differences. This hypothesis has been tested in animal models, in which ovariectomy of females eliminates, and treatment of ovariectomized (OVX) females with estradiol typically restores, this sex difference (Jackson et al., 2005; Lynch and Taylor, 2005; Ramôa et al., 2013). Yet despite this fairly extensive literature, little is known about the specific neural mechanisms underlying the effects of sex steroid hormones on female addiction.

The development and progression of addiction to drugs of abuse involves adaptations within the nucleus accumbens (NAc), a component of the mesolimbic reward pathway. These drug-induced changes in structural and functional plasticity are targeted toward medium spiny neurons (Dietz et al., 2009), the principal output neurons of the NAc. Similar to cocaine (Nazarian et al., 2008; Dumitriu et al., 2012), estradiol alters excitability (Mermelstein et al., 1996), gene expression (Grove-Strawser et al., 2010), and dendritic structure in medium spiny neurons (Staffend et al., 2011; Peterson et al., 2014). Consequently, estradiol may act in concert with drugs of abuse to induce plasticity within mesolimbic reward areas, thereby conferring increased susceptibility to the addictive effects of these drugs in females.

One mechanism whereby estradiol may enhance drug-induced plasticity is via interactions with group I metabotropic glutamate receptors (mGluRs). There is a growing body of evidence linking group I mGluRs, and in particular mGluR5, to responses to nicotine, alcohol, and psychostimulants (Pomierny-Chamioło et al., 2014). As a result, drugs that block activation of mGluR5 [e.g., 2-methyl-6-(phenylethynyl)pyridine hydrochloride (MPEP)] have been examined for their potential therapeutic effects on drug addiction (Kenny et al., 2003; Olive et al., 2005; Cozzoli et al., 2009; Kumaresan et al., 2009). Little effort, however, has been directed toward examining the effects of mGluR5 blockade on addiction in females. This is particularly surprising because estradiol activates mGluR5 signaling within the NAc core, leading to altered dendritic structure and enhancement of cocaine-mediated behavioral sensitization (Grove-Strawser et al., 2010; Martinez et al., 2014; Peterson et al., 2014). Hence, the present experiments sought to examine the role of mGluR5 signaling on cocaine intake under extended access conditions. In contrast to short access, extended access results in higher and more unstable cocaine intake patterns over time (Lynch and Taylor, 2005; Ramôa et al., 2013), which may be more reflective of an addicted phenotype. Given previous reports of enhancing effects of estradiol across several models of extended access to cocaine (Lynch and Taylor, 2005; Larson et al., 2007; Ramôa et al., 2013), here we tested the hypothesis that mGluR5 activation is necessary to mediate the facilitative effects of estradiol on cocaine intake in OVX female rats.

## Materials and Methods

### Animals

OVX female Sprague-Dawley rats were purchased at 8 weeks of age (175–199 g) from either Envigo (Indianapolis, IN) or Charles River Laboratories (Raleigh, NC; vendor controlled for across experiments) and pair-housed in polycarbonate cages with wire mesh tops. Animals were maintained on a 12:12-h light:dark cycle (lights on at 7 a.m.), with all behavior testing occurring between the hours of 8 a.m. and 2 p.m. Food and water were available *ad libitum*, with the exception that food was restricted during self-administration procedures. At these times, animals received 8–12 g of food to maintain body weight at approximately 90% of their initial *ad libitum* weight. Animal procedures were carried out in accordance with the *Guide for the Care and Use of Laboratory Animals* (8th edition) and approved by the University of Minnesota Institutional Animal Care and Use Committee.

### Surgery

Female rats were administered the analgesic carprofen (5 mg/ml/kg s.c.; 193.70200.3, Midwest Veterinary Supply, Lakeville, MN) and subsequently anesthetized with isoflurane (2.5–4% in oxygen; 193.33161.3, Midwest Veterinary Supply). A short segment of the right jugular vein was isolated and externalized, and a small incision was made into the vein to allow entry of a polyurethane jugular catheter (C30PU-RJV1405, Instech, Plymouth Meeting, PA). The indwelling end of the catheter was secured to the jugular vein using silk sutures. The free end of the catheter was routed subcutaneously such that it exited between the scapulae, and then was connected to an infusion harness (VAH95AB, Instech). Just before completion of surgery, animals were infused i.v. with 0.1 ml of the antibiotic enrofloxacin (22.7 mg/ml; 515.10010.3, Midwest Veterinary Supply) followed by 0.2 ml of heparinized saline (50 IU/ml; 191.46700.3, Midwest Veterinary Supply). Carprofen, enrofloxacin, and heparinized saline injections continued daily for the first three postoperative days. Catheters were flushed each morning with 0.2 ml of heparinized saline containing the antibiotic cefazolin (10 mg/ml; 191.31200.3, Midwest Veterinary Supply), beginning on the fourth postoperative day and continuing throughout the remainder of the experiment. Catheter patency was assumed if there was little or no resistance during daily catheter flushes. Animals exhibiting signs of edema were treated daily with 0.1–0.2 ml of furosemide (5 mg/ml; 193.22050.3, Midwest Veterinary Supply). All animals were allowed to recover for at least 1 wk before onset of cocaine self-administration training.

### Drugs

Estradiol (17β-estradiol; E2758, Sigma-Aldrich, St. Louis, MO) was dissolved in cottonseed oil to a final concentration of 2 μg/0.1 ml and was injected s.c. at a volume of 0.1 ml. The mGluR5 antagonist MPEP (1212, Tocris, Minneapolis, MN) was dissolved in sterile saline (1 mg/ml/kg) and injected i.p. This dose of MPEP has been shown to block estradiol-induced changes in dendritic spines within the NAc (Peterson et al., 2014), as well as estradiol enhancement of behavioral sensitization (Sircar and Kim, 1999; Martinez et al., 2014), in OVX female rats. The mGluR5-positive allosteric modulator 3-cyano-*N*-(1,3-diphenyl-1H-pyrazol-5-yl)benzamide (CDPPB; 3235, Tocris) was dissolved in 10% Tween-80 (low CDPPB, 10 mg/ml/kg; high CDPPB, 25 mg/2 ml/kg) and injected i.p. The 10 mg/ml/kg dose of CDPPB mimics the effects of estradiol on dendritic spine density in the NAc in OVX females (Gross et al., 2016). Cocaine (cocaine hydrochloride; 0406-1520-53, Mallinckrodt, St. Louis, MO) was dissolved in sterile PBS (9.3 mg/ml) and infused i.v. (1.5 mg/kg/infusion). Previous work has shown that under extended access conditions, estradiol treatment enhances cocaine intake in OVX females self-administering at this dose (Ramôa et al., 2013).

### Behavior

#### Testing apparatus

Self-administration behaviors were assessed in operant chambers housed within ventilated, sound-attenuating cubicles (Med Associates, St. Albans, VT). Each chamber was outfitted with an infusion tether connected to a swivel arm, a pellet dispenser/hopper, two levers, two stimulus lights (one above each lever), and a house light. Infusion tethers were connected to 20-ml syringes driven by infusion pumps. Pumps were positioned adjacent to (but outside) each sound-attenuating cubicle. Operant chambers were connected to a control box linked to a PC running MED-PC IV software (Med Associates).

#### Pellet self-administration training

Before undergoing jugular vein catheter surgery (experiments 1 and 2) or extended access pellet self-administration (experiment 3), animals were trained to self-administer 45 mg of chocolate-flavored sucrose pellets (F07256, Bio-Serv, Flemington, NJ) on a fixed ratio 1 (FR1) schedule. Daily, 6-h sessions began at approximately 8 a.m. During the sessions, each press of the right lever resulted in activation of the pellet hopper (and consequent dispensing of a single sucrose pellet into the food hopper), followed by a brief (1-s) timeout period. The associated stimulus light was activated throughout the dispensing and timeout periods. Additional pressing of the right lever during these periods was recorded but had no consequence. The left lever behaved identically to the right lever, with the exception that pressing it did not activate the pellet dispenser. Animals were allowed to self-administer a maximum of 100 pellets per day, at which point additional presses on either lever had no consequence. Animals were considered to have learned to self-administer sucrose pellets if they received the daily maximum of 100 pellets for three consecutive days. Any animal that failed to learn the task within 7 d was excluded from the study. Throughout this training task, all animals received injections of estradiol 30 min before testing on a 2 d on/2 d off schedule. This pattern of injections was chosen to mimic the cyclic changes in estradiol that occur across the 4-d estrous cycle of the female rat.

#### Cocaine self-administration training

One week after surgery, females were trained to self-administer cocaine on an FR1 schedule (1.5 mg/kg/infusion). Daily, 6-h sessions began at 8 a.m. These sessions were structured similarly to pellet self-administration training sessions, with the following exceptions. First, each press of the right lever resulted in activation of the syringe pump for approximately 2 s (1.7–2.3 s, depending on animal weight), followed by a 5-s timeout period. Second, animals were allowed to receive a maximum of 20 infusions of cocaine per day and were considered to have learned to self-administer cocaine if they received that maximum number of infusions on three consecutive days. Finally, no hormone injections were performed during cocaine self-administration training, given that estradiol is known to enhance acquisition of cocaine self-administration (Hu and Becker, 2008), and the current study sought to specifically examine the role of estradiol (and downstream signaling mechanisms) on intake under extended access conditions.

#### Extended access cocaine self-administration

At the conclusion of cocaine self-administration training, females continued to self-administer cocaine on an FR1 schedule (1.5 mg/kg/infusion) during daily 6-h sessions for a period of 10 consecutive days. In contrast to training sessions, there were no maximum daily infusion limits during this extended access period. Under these testing conditions, both male and female rats exhibit moderate-to-high levels of cocaine intake that typically does not increase over time, in comparison to using lower doses of cocaine, when escalation of intake normally occurs (Roth and Carroll, 2004; Lynch and Taylor, 2005; Wee et al., 2007; Ramôa et al., 2013). Given that cocaine intake is reliably higher in OVX, estradiol-treated females versus oil-treated controls when self-administering cocaine at 1.5 mg/kg/infusion (Lynch and Taylor, 2005; Ramôa et al., 2013), we chose to use this relatively high dose in the present study. For experiment 1, animals were injected i.p. with either MPEP or saline vehicle, followed by s.c. injections of estradiol or oil vehicle on a 2 d on/2 d off schedule for the duration of extended access. This resulted in four unique treatment conditions: oil plus saline (*n* = 12), oil plus MPEP (*n* = 10), estradiol plus saline (*n* = 9), and estradiol plus MPEP (*n* = 9). Injections occurred either 1 h (MPEP or saline) or 30 min (estradiol or oil) before testing. For experiment 2, animals were injected i.p. with a low dose of CDPPB (*n* = 8), a high dose of CDPPB (*n* = 6), or 10% Tween-80 vehicle (*n* = 10) 30 min before testing on a 2 d on/2 d off schedule for the duration of extended access.

#### Extended access pellet self-administration

After the completion of pellet training, a separate set of females (experiment 3) continued to self-administer 45 mg of chocolate-flavored sucrose pellets on a FR1 schedule during daily 6-h sessions for 10 consecutive days. This access schedule was designed to closely mimic the extended access cocaine self-administration protocol used in experiments 1 and 2. Consequently, there were no limits on the number of sucrose pellets that animals could receive during a 6-h session. Animals received s.c. injections of estradiol (*n* = 8) or oil vehicle (*n* = 7) on a 2 d on/2 d off schedule throughout the extended access period.

### Statistics

All data were analyzed using SPSS for Macintosh, version 23.0 (IBM, Armonk, NY). Data were first examined to determine whether the assumptions of parametric statistical tests were met. *p*-values of less than 0.05 were considered *a priori* to be significant. For experiment 1, the effects of drug (MPEP or vehicle), hormone (estradiol or vehicle), and time (day of extended access) on the number of drug infusions and inactive lever presses during extended access were examined via mixed-design factorial ANOVA. Statistically significant two-way interactions were further decomposed for the effect of hormone at each day via independent samples *t*-tests, and for the effect of hormone at each level of drug via mixed-design factorial ANOVA. For experiment 2, the effects of drug (low CDPPB, high CDPPB, or vehicle) and time on the number of drug infusions and inactive lever presses during extended access were examined via mixed-design factorial ANOVA. With statistically significant effects of time, individual sessions were compared via paired-samples *t*-tests (Holm adjustment to Bonferroni test for post-hoc comparisons). Statistically significant two-way interactions were further analyzed examining the effect of CDPPB on each test day via one-way ANOVA with Tukey’s honestly significant difference test for post hoc comparisons. For experiment 3, the effects of hormone (estradiol or vehicle) and the effects of time on the number of pellets received and the number of inactive lever presses were examined via mixed-design factorial ANOVA. Statistically significant effects of time were further explored as described for experiment 2. Data distributions and observed power are presented in Table 1 (superscripts associated with each analysis refer to table lines).

**Table 1.** Statistical analysis.

**Line**	**Data structure**	**Type of test**	**Observed power**
a	Normally distributed	ANOVA, mixed measures, repeated factor main effect	0.998
b	Normally distributed	ANOVA, mixed measures, interaction effect	0.882
c	Normally distributed	Independent samples *t*-test	0.053
d	Normally distributed	Independent samples *t*-test	0.669
e	Normally distributed	ANOVA, mixed measures, interaction effect	0.384
f	Normally distributed	ANOVA, mixed measures, interaction effect	0.585
g	Normally distributed	ANOVA, mixed measures, independent factor main effect	0.628
h	Normally distributed	ANOVA, mixed measures, independent factor main effect	0.081
i	Normally distributed	ANOVA, mixed measures, independent factor main effect	0.053
j	Normally distributed	ANOVA, mixed measures, repeated factor main effect	1.000
k	Normally distributed	ANOVA, mixed measures, interaction effect	1.000
l	Normally distributed	ANOVA, mixed measures, independent factor main effect	0.054
m	Normally distributed	ANOVA, mixed measures, repeated factor main effect	0.983
n	Normally distributed	ANOVA, mixed measures, independent factor main effect	0.053
o	Normally distributed	ANOVA, mixed measures, interaction effect	0.225

## Results

### Experiment 1: Estradiol facilitation of cocaine self-administration is dependent on mGluR5

**Figure 1.**
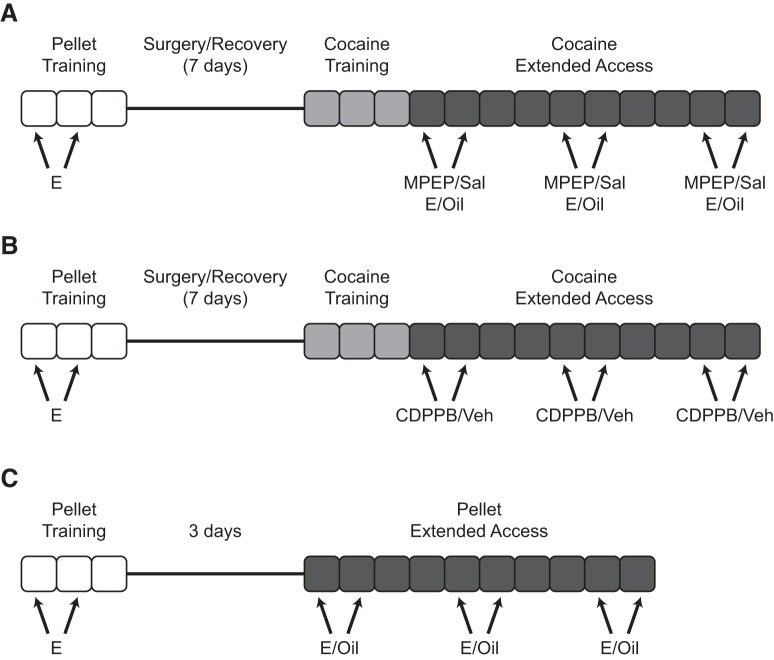
Timeline of experimental manipulations. ***A***, For experiment 1, ovariectomized female rats were first trained to self-administer sucrose pellets (pellet training) on a FR1 schedule during daily 6-h sessions (maximum 100 pellets/d). Animals then underwent implantation of jugular catheters, were allowed to recover, and were trained to self-administer cocaine (cocaine training) on an FR1 schedule during daily 6-h sessions (1.5 mg/kg/infusion; maximum 20 infusions/d). After training, animals were allowed to freely self-administer cocaine (cocaine extended access) for 10 consecutive days. All animals were injected s.c. with estradiol (E; 2 μg in 0.1 ml cottonseed oil) during pellet training; during extended access conditions, animals were injected i.p. with the mGluR5 antagonist MPEP (1 mg/ml/kg) or saline vehicle (Sal), followed 30 min later by s.c. injections of E or cottonseed oil vehicle (*n* = 9–12 per group). ***B***, Experiment 2 proceeded similarly to Experiment 1, with the exception that during extended access conditions, animals were injected i.p. with either the mGluR5 positive allosteric modulator CDPPB (low: 10 mg/ml/kg; high: 25 mg/2 ml/kg) or vehicle (Veh) (*n* = 6–10 per group). ***C***, For experiment 3, animals were trained as described in experiment 1, but then continued on to freely self-administer sucrose pellets (FR1 schedule; daily 6-h sessions) for 10 consecutive days (pellet extended access). Animals were injected s.c. with E or oil before testing (*n* = 7–8 per group).

**Figure 2.**
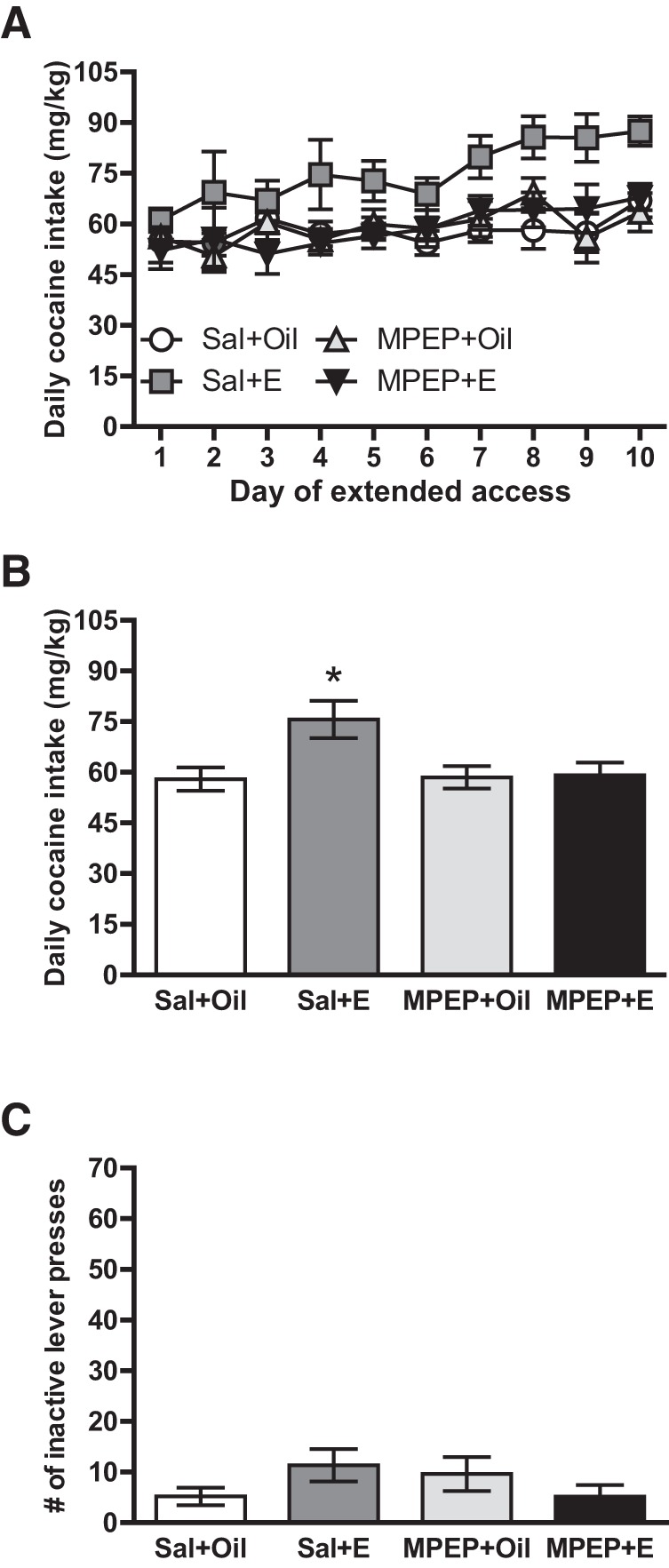
Mean (±SEM) responses during extended access conditions for experiment 1. ***A***, Although females in all treatment groups increased their cocaine intake across the 10 days of extended access, this effect was more pronounced in animals treated with estradiol (E) vs. oil. ***B***, When averaged across the extended access period, subjects pretreated with saline vehicle (Sal) before E had higher cocaine intake compared to oil-treated subjects, an effect that was not observed in subjects pretreated with the mGluR5 antagonist MPEP. In the absence of estradiol treatment, MPEP treatment alone did not significantly alter cocaine self-administration. *p < 0.05, Sal+E vs. Sal+Oil. ***C***, There were no significant effects of treatment on the number of inactive lever presses during extended access conditions.

We first tested the hypothesis that estradiol enhancement of extended access cocaine self-administration requires activation of mGluR5. To do so, we pretreated OVX females with the mGluR5 antagonist MPEP (or saline vehicle) 30 min before estradiol (or oil vehicle) and examined cocaine self-administration across 10 daily 6-h sessions (Fig. 1*A*). Subjects increased their cocaine intake over the extended access period, *F*(9,270) = 7.41, *p* < 0.001^a^ (Fig. 2*A*). This increase over time was more pronounced in estradiol- versus oil-treated subjects, *F* (9,270) = 2.162, *p* = 0.025^b^. Subjects treated with estradiol did not significantly differ from oil-treated subjects on d 1 of extended access, *t*(37) = –0.18, *p* = 0.86^c^, but by d 7, intake was significantly higher in estradiol-treated vs. oil-treated subjects, *t*(37) = –2.46, *p* = 0.019^d^. This pattern of elevated intake across hormone treatment groups continued through the remaining days of extended access. Whereas the three-way interaction of time × hormone × drug treatment condition was not statistically significant, *F*(2,270) = 0.78, *p* = 0.64^e^, there was a significant two-way interaction of hormone × drug treatment condition, *F*(1,30) = 5.05, *p* = 0.032^f^ (Fig. 2*B*). For saline-treated subjects, estradiol treatment resulted in significantly higher average daily cocaine intake vs. oil, *F*(1,16) = 5.93, *p* = 0.027^g^. In contrast, there was no significant difference between estradiol and oil treatment in subjects pretreated with MPEP, *F*(1,14) = 0.31, *p* = 0.59^h^. Importantly, MPEP treatment alone did not significantly alter daily intake in the absence of estradiol treatment, *F*(1,16) = 0.025, *p* = 0.88^i^. There were no significant main or interaction effects of drug or hormone on the number of inactive lever presses during extended access conditions (Fig. 2*C*).

### Experiment 2: mGluR5 activation is not sufficient to mimic the effects of estradiol on cocaine self-administration

**Figure 3.**
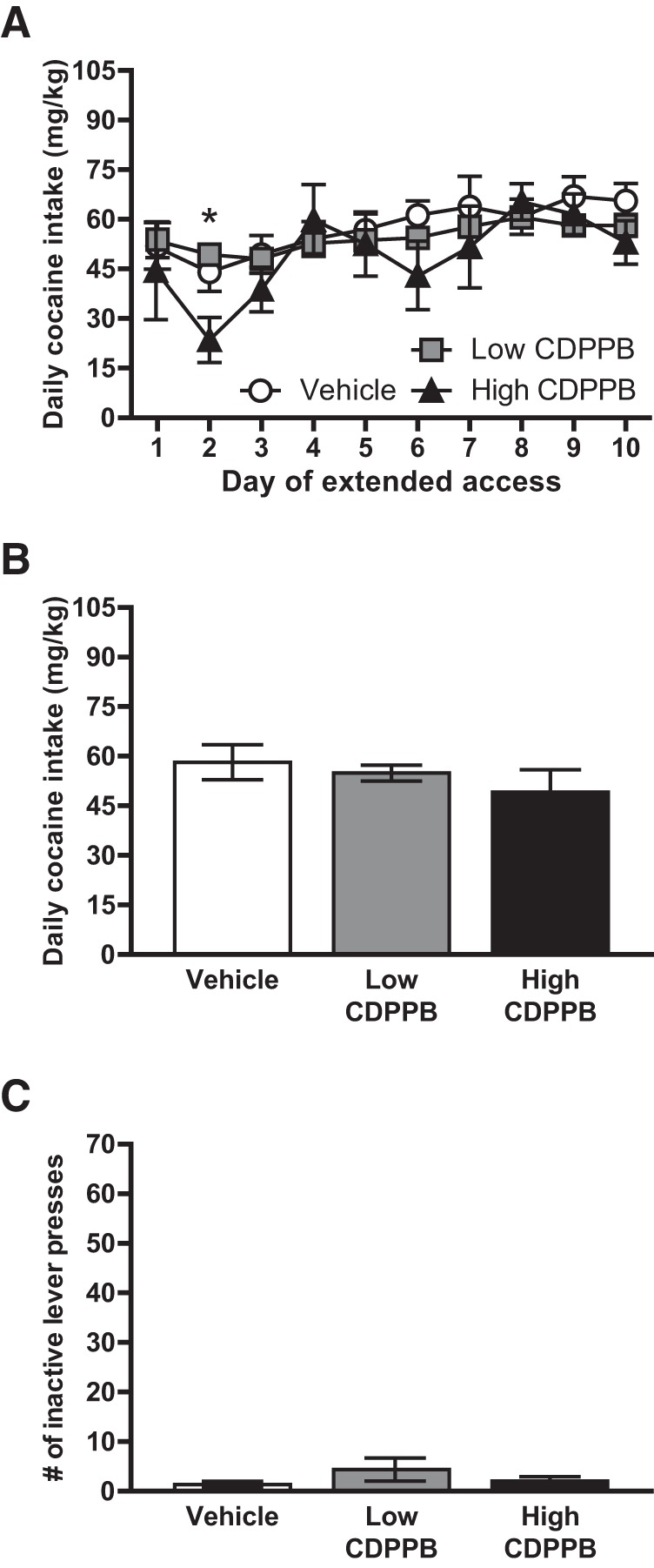
Mean (±SEM) responses during extended access conditions for experiment 2. ***A***, The effect of CDPPB treatment varied across sessions, with a significant decrease in intake observed in animals treated with the high dose of CDPPB versus either the low dose or 10% Tween-80 (vehicle) in session 2. *p < 0.05, high CDPPB vs. low CDPPB or vehicle. ***B***, ***C***, There were no significant effects of CDPPB treatment on cocaine intake (***B***) or inactive lever presses (***C***) when responses were averaged across sessions.

Given that blockade of mGluR5 via MPEP eliminated the facilitative effects of estradiol on cocaine self-administration, we next sought to determine whether stimulation of mGluR5 alone (in the absence of estradiol treatment) would be sufficient to drive enhanced self-administration in females. To address this question, OVX female rats were injected with the mGluR5-positive allosteric modulator CDPPB (low or high dose) or vehicle, 30 min before testing for cocaine self-administration under extended access conditions (Fig. 1*B*). These injections were administered on a 2 d on/2 d off schedule to mimic the estradiol treatment schedule used in experiment 1. Subjects altered their cocaine intake across the duration of the extended access period, *F*(9,153) = 17.506, *p* < 0.001^j^ (Fig. 3*A*). This effect was due to increases in cocaine intake by d 6–10 of extended access, compared with either d 2 or 3 of access (Holm modification to the Bonferroni test for post hoc comparisons). The change in intake over time varied significantly across drug treatment conditions (low CDPPB, high CDPPB, or vehicle), *F*(18,153) = 3.369, *p* < 0.001^k^. On d 2 of extended access, subjects treated with high CDPPB had significantly reduced intake versus either low CDPPB– or vehicle-treated females (Tukey’s honestly significant difference test for post hoc comparisons). No significant differences between drug treatment groups were observed on any other test day (all *p* > 0.05). Irrespective of time, there was no main effect of CDPPB on cocaine intake, *F*(2,17) = 0.30, *p* = 0.97^l^ (Fig. 3*B*). Finally, there was no effect of drug on the number of inactive lever responses (Fig. 3*C*).

### Experiment 3: Estradiol does not alter self-administration of sucrose pellets under extended access conditions

**Figure 4.**
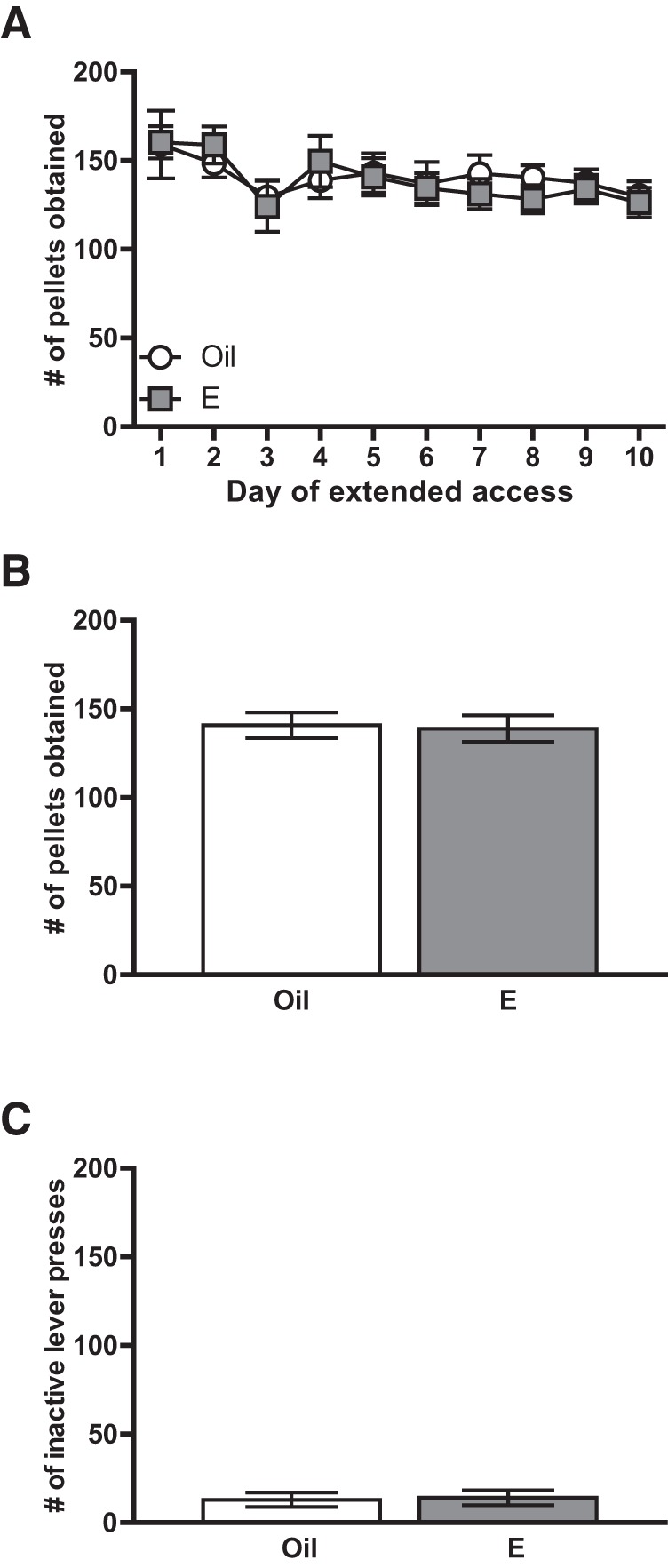
Mean (± SEM) responses during extended access conditions for experiment 3. ***A***, The number of sucrose pellets obtained by females during daily sessions decreased over time, irrespective of treatment with estradiol (E) or oil. ***B***, ***C***, There were no significant effects of estradiol treatment on the number of pellets obtained (***B***) or inactive lever presses (***C***) when responses were averaged across sessions.

To determine whether the effects of experiment 1 (i.e., estradiol enhancement of cocaine self-administration) generalize to nondrug rewards, an additional experiment was conducted examining sucrose pellet self-administration. OVX female rats were injected with estradiol (or oil vehicle) and then tested for sucrose pellet self-administration across 10 daily 6-h sessions (Fig. 1*C*). There were statistically significant effects of time on the number of sucrose pellets obtained, *F*(9,117) = 3.449, *p* < 0.001^m^ (Fig. 4*A*). This effect was reflected in a tendency of the number of pellets obtained to decrease over sessions, but was only significant for the comparison of the 1st versus 6th sessions (Holm adjustment to Bonferroni test for post hoc comparisons). Notably, there were no significant effects of hormone (estradiol vs. oil), *F*(1,13) = 0.03, *p* = 0.86^n^, or time × hormone interaction, *F*(9,117) = 0.48, *p* = 0.89^°^, on the number of sucrose pellets obtained (Fig. 4*A*, *B*). Additionally, there was no significant effect of hormone on the number of inactive lever responses (Fig. 4*C*).

## Discussion

The data presented here indicate that estradiol enhancement of cocaine self-administration in OVX female rats depends critically on activation of mGluR5. In contrast, allosteric mGluR5 potentiation alone is insufficient to mimic the effects of estradiol on this behavior. Finally, the effects of estradiol (and associated downstream mechanisms) on cocaine intake appear fairly specific to drug-related behaviors, since estradiol treatment did not enhance self-administration of nondrug (i.e., food) rewards. When considered in the context of previous research linking mGluR5 in estradiol facilitation of cocaine-induced behavioral sensitization in OVX females (Martinez et al., 2014), these data implicate mGluR5-dependent signaling as a critical mechanism whereby estradiol enhances responses to cocaine in this sex.

The enhanced response of women to drugs of abuse is a well-established sex difference in addiction (Greenfield et al., 2010). To our knowledge, the present experiments are the first to date examining the role of mGluR5 during extended access to cocaine in either males or females. Previous work in males has demonstrated that acute treatment with the mGluR5 antagonist MPEP decreases cocaine intake in rats that had already established patterns of cocaine intake (Tessari et al., 2004; Kenny et al., 2005). Our study complements and extends those findings, by demonstrating that MPEP treatment disrupts the enhancement of cocaine intake normally induced by estradiol in OVX females tested under extended access conditions. It should be noted that in contrast to previous studies in males, MPEP treatment alone did not affect cocaine intake in these females. This was not surprising, however, given that this dose of MPEP (1 mg/kg) also fails to affect behavioral sensitization in OVX females (Martinez et al., 2014).

The brain areas wherein mGluR5 activation is necessary for estradiol enhancement of cocaine self-administration were not directly examined in the present study. However, previous studies implicate the NAc as a likely candidate. In males, this brain area is known to regulate cocaine self-administration (Zito et al., 1985), and cocaine intake under extended access conditions results in dysregulated mGluR5 expression within the NAc (Hao et al., 2010). Although site-specific manipulations of mGluR5 combined with extended access cocaine self-administration have not been performed in either sex, evidence from studies of males examining other aspects of cocaine seeking, including reinstatement following forced withdrawal, implicate the core subdivision of the NAc (NAcC). Specifically, in males, blockade of mGluR5 in the NAcC decreases cocaine seeking in cue-, context-, and cocaine-induced reinstatement testing (Wang et al., 2013; Knackstedt et al., 2014), whereas activation of mGluR5 in the NAcC enhances cue-induced reinstatement of cocaine seeking (Wang et al., 2013). These studies raise the intriguing possibility that estradiol may coopt existing mGluR5 machinery in the NAcC that is present in both sexes, ultimately providing an additional drive on this system to enhance responses to drugs of abuse in females. Indeed, the NAcC is the only known brain region in which membrane estrogen receptors (i.e., ERα) sex-specifically activate mGluR5 and directly affect synaptic structure (Grove-Strawser et al., 2010; Peterson et al., 2014). In various other brain regions, estradiol-group I mGluR signaling occurs via mGluR1a (Boulware et al., 2005, 2013; Dewing et al., 2007; Christensen et al., 2011; Huang and Woolley, 2012). Notably, estradiol can also influence nervous system structure/function through a wide range of mGluR-independent mechanisms, including activation of estradiol-sensitive G-protein–coupled estrogen receptors and, of course, nuclear estrogen receptors (Micevych and Dominguez, 2009). In addition, mGluR5 can clearly function independently of estradiol in females. The effects of estradiol on plasticity in the NAc of OVX females are mediated by mGluR5 in the NAcC, but not in the shell subdivision; MPEP treatment alone has no effect in either subdivision (Peterson et al., 2014). In contrast, treatment of OVX females with CDPPB (in the absence of estradiol) induces plasticity in both regions (Gross et al., 2016). It is perhaps not surprising, then, that widespread activation of mGluR5 (via systemic CDPPB administration) did not mimic the effect of estradiol in the present study. The transient decrease in cocaine intake observed after CDPPB administration could represent effects of CDPPB in areas of the brain wherein estradiol-mGluR5 signaling does not occur, in line with the effects of CDPPB on structural plasticity described above. Additional studies involving site-specific activation of mGluR5 will be required to determine whether local activation of this receptor can exert differential/competing effects on responses to cocaine in females.

ERα/mGluR5 signaling can rapidly induce a sequence of signaling events that may be critical for the development of an addicted phenotype. Estradiol induces dopamine release in the striatum via disinhibition of local dopaminergic terminals (Becker, 1990; Thompson and Moss, 1994; Hedges et al., 2010), an effect that is mediated by classic estrogen receptors (Xiao et al., 2003) and mimicked by activation of group I mGluRs (Bruton et al., 1999). The effects of estradiol on dopamine release specifically within the NAc can be fairly rapid and transient (Thompson and Moss, 1994) and may not always be observed when dopamine is sampled along longer time frames (Cummings et al., 2014). One mechanism that may link ERα/mGluR5 signaling to changes in dopamine release is the endogenous endocannabinoid system. Within the hippocampus, estradiol rapidly suppresses GABAergic signaling (Murphy et al., 1998), an effect that is dependent on both group I mGluR and endocannabinoid signaling and is specific to females (Huang and Woolley, 2012). Although similar effects of estradiol have not yet been demonstrated in the NAc, GABAergic medium spiny neurons (the principle output cell of the dorsal/ventral striatum) express ERα (Almey et al., 2016), and activation of cannabinoid receptor subtype 1 in the NAc rapidly induces dopamine release (Sperlágh et al., 2009). Recent work extends these findings by demonstrating that estradiol enhancement of behavioral sensitization to cocaine in females is prevented by blockade of cannabinoid receptor subtype 1 (Peterson et al., 2016). Considered together, these data suggest that the endogenous endocannabinoid system may be a crucial link between ERα/mGluR5 signaling in the NAc and the development/expression of addictive behaviors in females.

Estradiol signaling through ERα/mGluR5 has very rapid (on the order of seconds/minutes) effects on neuronal excitability (Grove-Strawser et al., 2010), followed by slower (on the order of hours/days) effects on dendritic spine plasticity (Peterson et al., 2014). This parallels what is observed in other systems, including the hypothalamus. In this system, estradiol signaling via ERα/mGluR1a leads to a rapid internalization of μ-opioid receptors in the medial preoptic area (Dewing et al., 2007), followed by a slower, lasting increase in dendritic spine density in the arcuate nucleus (Christensen et al., 2011). Intriguingly, both the slower and the more rapid effects of estradiol within the hypothalamus are required for the normal expression of sexual receptivity in females (Kow and Pfaff, 2004). It stands to reason, then, that both the rapid effects of ERα/mGluR5 signaling on neuronal excitability and the slower effects of this signaling pathway on dendritic spine plasticity, may work synergistically within the NAc to enhance motivated behaviors in females. This idea would seem to be supported by our finding that differences in the number of cocaine infusions between estradiol- and oil-treated females did not become evident until 6 d after their first estradiol injection.

In summary, our data suggest that estradiol acts via an mGluR5-dependent mechanism to enhance cocaine self-administration in OVX female rats. Given the existing literature implicating mGluR5 in responses to drugs of abuse in males, these data provide further support for the therapeutic potential of pharmacological agents that block the effects of mGluR5, including MPEP. Perhaps more importantly, linking the addiction-enhancing effects of estradiol to the intracellular signaling pathways associated with group I mGluRs opens up a range of potential therapeutic targets beyond mGluR5, which may prove particularly valuable in the development of more effective treatments for addiction in women.

## Synthesis

The decision was a result of the Reviewing Editor Karen Szumlinski and the peer reviewers coming together and discussing their recommendations until a consensus was reached. A fact-based synthesis statement explaining their decision and outlining what is needed to prepare a revision is listed below. The following reviewer(s) agreed to reveal their identity: Jill Becker

After consultation with the original 2 expert reviewers, we all agree that the report is much improved. Both reviewers had 1 minor remaining concern each, that I would like to see incorporated into the text prior its final acceptance. As such, I am issuing a decision of Revise-Editorial Review only and I look forward to receiving the final version of the report soon.

Reviewer #1:

I commend the authors on appropriately responding to my prior concerns. One minor comment (for which I will defer to the Editor's final decision) is that the lack of escalation might be worthy of comment. In particular, that high dose infusions are associated with low (or perhaps negligible) escalation which is also reported by Wee et al J Pharmacol Exp Ther. 2007 Mar;320(3):1134-43.

Reviewer #2:

This is an improved manuscript. The results are clear and the discussion is very helpful for putting the results into perspective. I have only one request - on page 13, line 293, please insert 'in males' after 'Specifically' to make that sentence clear should it be taken or read out of context.
